# Supplementary material for: Comparative Analysis of Tetra(2-naphthyl)tetracyano-porphyrazine and Its Iron Complex as Photosensitizers for Anticancer Photodynamic Therapy
Source: Pharmaceutics. 2022 Nov 30;14(12):2655. doi: 10.3390/pharmaceutics14122655 (PMC9786040; doi:10.3390/pharmaceutics14122655)
Supplement: Supplementary file 1 [file pharmaceutics-14-02655-s001.zip › pharmaceutics-1993521-supplementary.pdf]

Supplementary materials

Comparative Analysis of Tetra(2-naphthyl)tetracyanoporphyrazine and Its Iron Complex as Photosensitizers for Anticancer Photodynamic Therapy

Lydia N. Shestakova, Tatyana S. Lyubova, Svetlana A. Lermontova, Artem Artem O. Belotelov, Nina N. Peskova, Larisa G. Klapshina, Irina V. Balalaeva and Natalia Y. Shilyagina

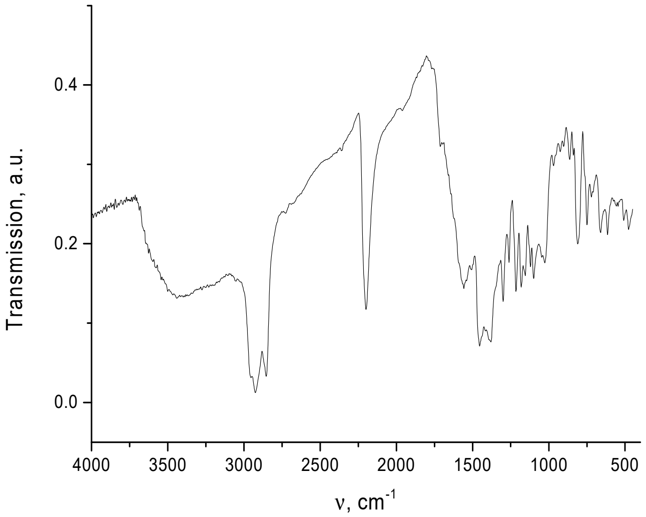

Figure S1. IR spectrum of **FePzNPh** in mineral oil suspensions.

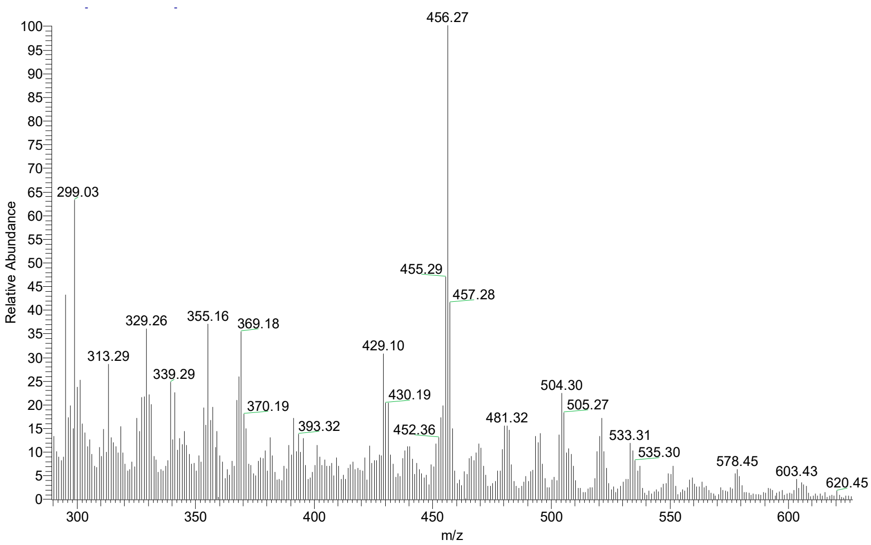

Figure S2. Mass-spectrum of **FePzNPh** at 70 eV in the mass number range of 50–1000.

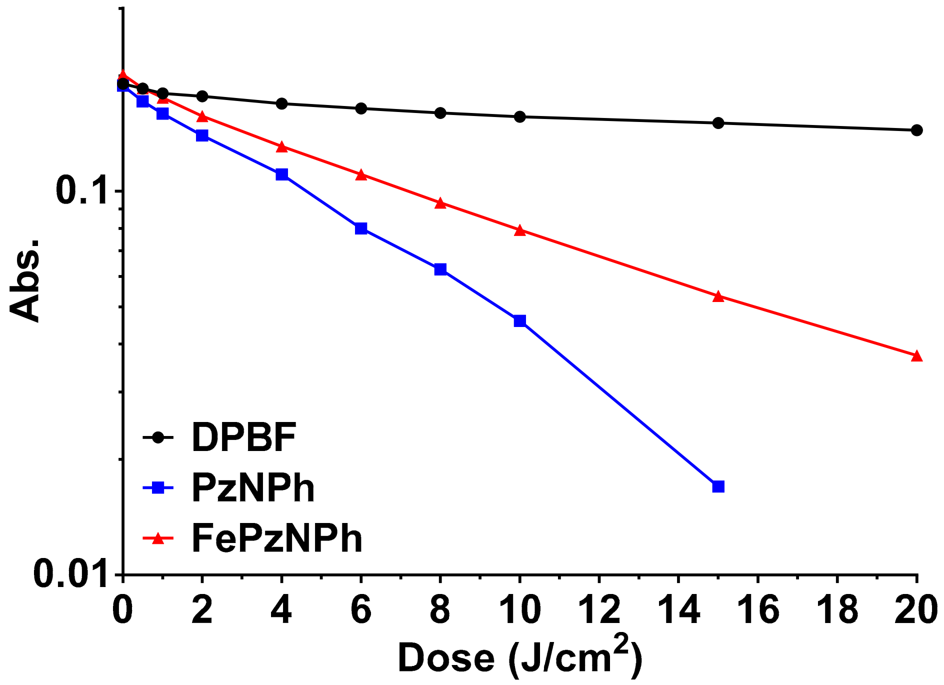

Figure S3. Rate of decay of DPBF sensitized by **PzNPh** and **FePzNPh** in ethanol-glycerol mixture (50%) as shown by the decrease in the absorbance at 420 nm. To induce a photodynamic response, the cells were irradiated with light (615–635 nm, 20 mW/cm^2).
